# Time-dose reciprocity mechanism for the inactivation of *Escherichia coli* explained by a stochastic process with two inactivation effects

**DOI:** 10.1038/s41598-022-26783-x

**Published:** 2022-12-30

**Authors:** Takahiro Matsumoto, Ichiro Tatsuno, Yukiya Yoshida, Makoto Tomita, Tadao Hasegawa

**Affiliations:** 1 Graduate School of Medical Sciences, Nagoya City University, Nagoya, 467-8601 Japan; 2 Graduate School of Design and Architecture, Nagoya City University, Nagoya, 464-0083 Japan; 3 Department of Physics, Faculty of Science, Shizuoka University, Shizuoka, 422-8529 Japan

**Keywords:** Biophysics, Microbiology

## Abstract

There is a great demand for developing and demonstrating novel disinfection technologies for protection against various pathogenic viruses and bacteria. In this context, ultraviolet (UV) irradiation offers an effective and convenient method for the inactivation of pathogenic microorganisms. The quantitative evaluation of the efficacy of UV sterilization relies on the simple time-dose reciprocity law proposed by Bunsen-Roscoe. However, the inactivation rate constants reported in the literature vary widely, even at the same dose and wavelength of irradiation. Thus, it is likely that the physical mechanism of UV inactivation cannot be described by the simple time-dose reciprocity law but requires a secondary inactivation process, which must be identified to clarify the scientific basis. In this paper, we conducted a UV inactivation experiment with *Escherichia coli* at the same dose but with different irradiances and irradiation durations, varying the irradiance by two to three orders of magnitude. We showed that the efficacy of inactivation obtained by UV-light emitting diode irradiation differs significantly by one order of magnitude at the same dose but different irradiances at a fixed wavelength. To explain this, we constructed a stochastic model introducing a second inactivation rate, such as that due to reactive oxygen species (ROS) that contribute to DNA and/or protein damage, together with the fluence-based UV inactivation rate. By solving the differential equations based on this model, the efficacy of inactivation as a function of the irradiance and irradiation duration under the same UV dose conditions was clearly elucidated. The proposed model clearly shows that at least two inactivation rates are involved in UV inactivation, where the generally used UV inactivation rate does not depend on the irradiance, but the inactivation rate due to ROS does depend on the irradiance. We conclude that the UV inactivation results obtained to date were simply fitted by one inactivation rate that superimposed these two inactivation rates. The effectiveness of long-term UV irradiation at a low irradiance but the same dose provides useful information for future disinfection technologies such as the disinfection of large spaces, for example, hospital rooms using UV light, because it can reduce the radiation dose and its risk to the human body.

## Introduction

There is a great demand for developing and demonstrating efficient disinfection technologies to protect against various pathogenic viruses and bacteria. In this situation, sterilization by ultraviolet (UV) irradiation is attracting special interest because UV irradiation offers an effective and convenient method for the inactivation of pathogenic microorganisms, including coronaviruses^1–5^.

The principle of sterilization relies on the time-dose reciprocity law proposed by Bunsen-Roscoe^6^, Log(N/N_0_) = − Γ × D, where Γ (cm^2^/mJ) is the inactivation rate constant depending on the wavelength, D = τ × P, D (mJ/cm^2^) is the UV dose, P (mW/cm^2^) is the UV irradiance and τ (s) is the irradiation duration (Hereafter, we use D as UV dose, P as UV irradiance, and τ as irradiation duration.). This reciprocity law has been applied to many different categories of photoreaction processes, such as photopolymerization, photoconductance, and photodegradation, as well as UV sterilization^7^. The reciprocity law assumes that the rate of the photochemical reaction process is proportional to the light irradiance (linear stochastic process) such that the amount of the process depends only on the D. While this is true for most primary photochemical reaction processes at light irradiances which do not induce nonlinear effects, there are many reactions that do not obey the reciprocity law over any significant range of reaction conditions, such as radical polymerizations^8^. Furthermore, the inactivation rate constants of many bacteria and viruses by UV irradiation reported in the literature vary widely, even for studies in which the same wavelength of irradiation and the same types and strains of bacteria and viruses were used^9–11^. These wide range of reported values seem to suggest that the physical mechanism of UV inactivation cannot be described by the simple time-dose reciprocity law; instead, a secondary inactivation process must be identified to clarify the scientific basis^7^.

In this paper, we propose a stochastic model to explain the various inactivation rate constants for the same D. To validate the stochastic model proposed here, we conducted a UV inactivation experiment at the same D but different Ps and τs at a fixed wavelength, varying the P by two to three orders of magnitude. Here, we used *Escherichia coli* (*E. coli*) as an inactivation sample because this bacterium is one of the standard samples used to date in UV inactivation experiments^12–19^. The results obtained here show that at 265 nm, the efficacy of inactivation is greater for longer τs with a lower P at the same D. However, this tendency was less pronounced at 280 nm, and we did not observe such a significant difference at the irradiation wavelength of 308 nm.

To explain the efficacy of inactivation as a function of P and τ under the same D conditions, we obtained two sets of stochastic differential equations in which an inactivation rate [such as that due to reactive oxygen species (ROS)] that contributes to DNA and/or protein damage was introduced together with the conventional UV inactivation rate. By numerically solving the differential equations based on this model, the efficacy of inactivation as a function of the P and τ for the same D can be clearly explained. The proposed model clearly shows that at least two inactivation rates are involved in UV inactivation, where the generally used UV inactivation rate does not depend on the P, but the other rate does. Our conclusion suggests that the UV inactivation results obtained to date were simply fitted by one inactivation rate that superimposed these two inactivation rates.

## Materials and methods

### Culturing and counting of microorganisms

A pure culture of *E. coli* strain O1 was incubated in nutrient broth (E-MC63; EIKEN Chemical Co., Japan) at 37 °C for 20 h. A concentration of 10^9^ to 10^11^ colony forming units (CFU)/mL was obtained and used for the experiments. The pure culture of *E. coli* in the stationary phase was taken and diluted with a normal saline solution (9 g NaCl dissolved in 1 L purified water) to 10^3^ to 10^5^ colony forming units (CFU)/mL. To perform the inactivation experiments using UV-LEDs, 600 μL of the dispersed solution was taken and injected into a microtube. After the inactivation experiments, 100 μL of bacterial cells was taken and dispersed on agar plates. Colonies were counted after incubation for 24 h at 37 °C. The number of CFU/mL in the control suspension (without UV irradiation in the ultrasonic bath) was adjusted so that the number of CFUs in a plate after UV irradiation is in the range of 10^2^. For counting CFUs in the range of 10^1^ to 10^4^, we took a digital image of the plate, and we used Processing software (https://processing.org/) for the calculation of CFUs.

### UV LED characteristics and irradiation setup

**Figure 1:**
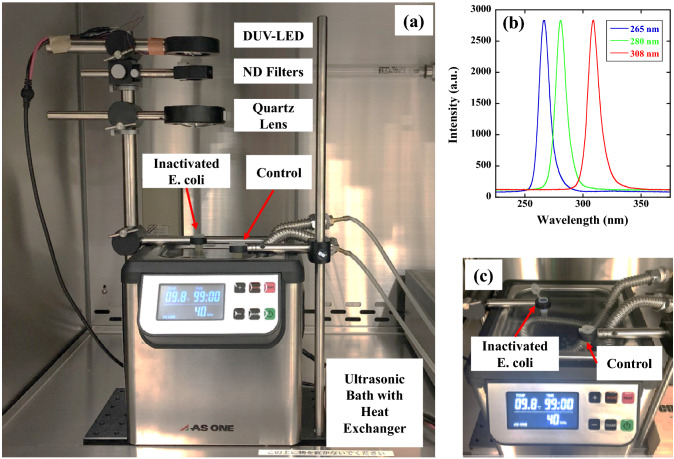
Optical setup of the UV inactivation system and emission spectrum of UV-LEDs. (**a**) Optical setup of the UV-LED inactivation system. (**b**) Emission spectra of 265 nm, 280 nm, and 308 nm UV-LEDs. These LEDs exhibited peak wavelength emissions at 266.5 nm, 280.6 nm, and 308.8 nm, respectively, with full-width at half-maximum bandwidths of 11.1 nm, 11.5 nm, and 12.0 nm. (**c**) Photograph of an *E. coli* bacterial sample irradiated by the UV-LED in an ultrasonic bath. An *E. coli* bacterial sample without UV irradiation was also placed in the ultrasonic bath as a control sample to account for the inactivation caused by ultrasonic vibrations.

Figure 1a shows the irradiation setup for the inactivation system. UV-exposure experiments were conducted using 265, 280, or 308 nm UV LEDs (265 nm: 265-FL-01-G01, 280 nm: 280-FL-01-G01, and 308 nm: 308-FL-01-G01, DOWA ELECTRONICS MATERIALS CO., LTD., Japan). The UV spectra of the UV-LED wavelengths used in this condition (265 nm, 280 nm and 308 nm) were measured using a spectrometer through an optical fibre (BIM-6002A, Brolight Technology Corporation, Hangzhou, China). As shown in Fig. 1b, the 265 nm, 280 nm and 308 nm UV-LEDs exhibited peak wavelength emission at 266.5 nm, 280.6 nm and 308.8 nm, respectively, with full-width at half-maximum bandwidths of 11.1 nm, 11.5 nm and 12.0 nm. For the UV inactivation experiments, the UV irradiance was varied by the combination of UV-NIR neutral-density (ND) filters (#88-369, Edmund Optics Japan Ltd., Tokyo, Japan) whose optical density (OD) was varied from 0.3 to 3.5. We note here that we did not significantly change the applied voltage to the UV LEDs to obtain the same Ps but used them at around their rated voltages to prevent spectral peak shifts caused by changing the applied voltage. Furthermore, the transmission of UV-NIR ND filters changes significantly between 200 and 300 nm. Therefore, for the UV exposure experiments, different magnitudes of P were used as shown in Table 1.

**Table 1:** Combinations of irradiation duration τ (s) and irradiance P (mW/cm^2^) used to obtain the inactivation ratios plotted in Fig. 2.

(a) τ × P = 5 mJ/cm^2^ (265 nm)		(b) τ × P = 10 mJ/cm^2^ (265 nm)		(c) τ × P = 5 mJ/cm^2^ (280 nm)		(d) τ × P = 10 mJ/cm^2^ (280 nm)		(e) τ × P = 5 mJ/cm^2^ (308 nm)		(f) τ × P = 10 mJ/cm^2^ (308 nm)	
τ (s)	P (mW/cm^2^)	τ (s)	P (mW/cm^2^)	τ (s)	P (mW/cm^2^)	τ (s)	P (mW/cm^2^)	τ (s)	P (mW/cm^2^)	τ (s)	P (mW/cm^2^)
1	5.0E + 00	2	5.0E + 00	1	5.0E + 00	2	5.0E + 00	1	5.0E + 00	1	1.0E + 01
2	2.5E + 00	3	3.3E + 00	3	1.7E + 00	3	3.3E + 00	2	2.5E + 00	2	5.0E + 00
4	1.3E + 00	7	1.4E + 00	5	1.0E + 00	4	2.5E + 00	3	1.7E + 00	3	3.3E + 00
5	1.0E + 00	13	7.7E−01	15	3.3E−01	6	1.7E + 00	4	1.3E + 00	4	2.5E + 00
8	6.3E−01	27	3.7E−01	18	2.8E−01	12	8.3E−01	6	8.3E−01	6	1.7E + 00
16	3.1E−01	62	1.6E−01	36	1.4E−01	18	5.6E−01	12	4.2E−01	11	9.1E−01
31	1.6E−01	82	1.2E−01	71	7.0E−02	37	2.7E−01	16	3.1E−01	16	6.3E−01
102	4.9E−02	179	5.6E−02	92	5.4E−02	70	1.4E−01	32	1.6E−01	33	3.0E−01
114	4.4E−02	227	4.4E−02	140	3.6E−02	71	1.4E−01	59	8.5E−02	55	1.8E−01
207	2.4E−02	410	2.4E−02	157	3.2E−02	141	7.1E−02	66	7.6E−02	60	1.7E−01
288	1.7E−02	530	1.9E−02	211	2.4E−02	157	6.4E−02	111	4.5E−02	114	8.8E−02
380	1.3E−02	666	1.5E−02	307	1.6E−02	180	5.6E−02	123	4.1E−02	122	8.2E−02
398	1.3E−02	980	1.0E−02	345	1.4E−02	206	4.9E−02	178	2.8E−02	175	5.7E−02
569	8.8E−03	1052	9.5E−03	402	1.2E−02	314	3.2E−02	229	2.2E−02	209	4.8E−02
656	7.6E−03	1648	6.1E−03	431	1.2E−02	421	2.4E−02	243	2.1E−02	246	4.1E−02
942	5.3E−03	1909	5.2E−03	775	6.5E−03	430	2.3E−02	323	1.5E−02	318	3.1E−02
1159	4.3E−03	2126	4.7E−03	1023	4.9E−03	613	1.6E−02	350	1.4E−02	353	2.8E−02
1367	3.7E−03	2392	4.2E−03	1231	4.1E−03	804	1.2E−02	493	1.0E−02	459	2.2E−02
2507	2.0E−03	3670	2.7E−03	1947	2.6E−03	861	1.2E−02	636	7.9E−03	615	1.6E−02
		4387	2.3E−03			1224	8.2E−03	706	7.1E−03	683	1.5E−02
						1265	7.9E−03	1122	4.5E−03	902	1.1E−02
						1627	6.1E−03	1256	4.0E−03	1236	8.1E−03

Then, after transmission through ND filters, the UV radiation was collimated by using a convex lens with a focal length of 80 mm and was guided to a microtube made of polypropylene (2-8007-02, AS ONE Corporation, Osaka, Japan) that contained a suspension of *E. coli* (600 μL). The obtained beam diameter was approximately 20 mm in diameter, and the diameter of the microtube was 9 mm; therefore, the whole region of the suspension was irradiated by UV radiation. The P of UV radiation to which the bacteria were subjected was measured each time by placing a UV-extended Si photodiode with an aperture of 9.5 mm (S120VC, Thorlabs Inc., New Jersey, USA) on the surface of the microtube. Based on this measured value, the τ was determined. The combinations of P and τ at each irradiation wavelength are listed in Table 1.

The suspension in the tube was homogeneously diffused by using an ultrasonic bath (MCS-2, AS ONE Corporation, Osaka, Japan) with a frequency of 40 kHz and an output power of 55 W. The temperature of the ultrasonic bath was maintained at 23 °C by using a heat exchanger, which inhibited the temperature increase caused by 60 min of ultrasonic operation. The temperature of the microtube would have risen to approximately 50 °C throughout the 60 min of ultrasonic operation (not due to UV irradiation) without the heat exchanger. The control suspension, which was not subjected to UV irradiation, was also placed in the ultrasonic bath at every measurement to precisely distinguish and exclude the inactivation caused by ultrasonication from that caused by UV irradiation, as shown in Fig. 1c. We note here that the CFU reduction caused by 60 min of ultrasonication was less than 10% of the CFU of the initial control sample^20–22^.

### Statistical analysis

The log inactivation was calculated as Log (N/N_0_) with base 10, where N is the CFU number after UV irradiation in the ultrasonic bath, and N_0_ is the CFU number without UV irradiation in the ultrasonic bath. This procedure was performed in every experiment. All experiments were performed at least three times independently. All data were expressed as the mean ± standard deviation. The statistical analyses of the data were performed using a paired Student’s *t* test. The *p*-values < 0.05 were considered statistically significant.

## Results and analysis

### Inactivation of *E. coli* at the same dose but different irradiances at the fixed wavelength condition

**Figure 2:**
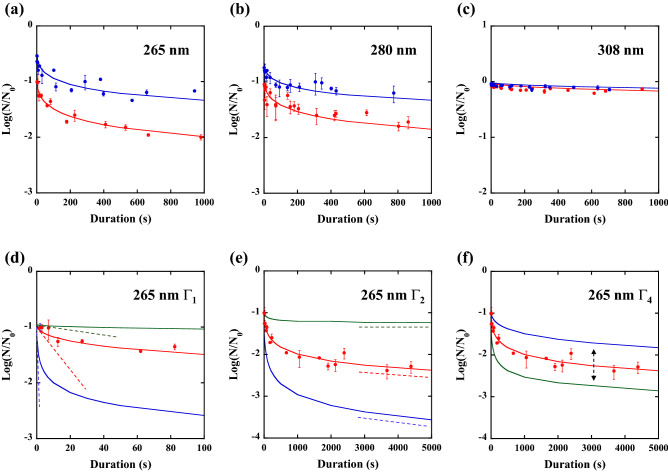
Experimental plots of the inactivation ratio [Log(N/N_0_)] for various irradiation durations (and various irradiances) at doses of 10 mJ/cm^2^ (red circles) and 5 mJ/cm^2^ (blue circles) and irradiation wavelengths of (**a**) 265 nm, (**b**) 280 nm, and (**c**) 308 nm. The red or blue line represents the theoretically fitted inactivation ratio as a function of irradiation duration at a constant dose (red: 10 mJ/cm^2^, blue: 5 mJ/cm^2^) but different irradiance conditions. (**d**) Determination of Γ_1_ by the initial slope of the curve for 265 nm and 10 mJ/cm^2^ results, where the green curve is described by Γ_1_ = 2 × 10^−4^ cm^3^/s, the red curve is described by Γ_1_ = 2 × 10^−3^ cm^3^/s, and the blue curve is described by Γ_1_ = 2 × 10^−2^ cm^3^/s. (**e**) Determination of Γ_2_ by the tail slope of the curve for 265 nm and 10 mJ/cm^2^ results, where the green curve is described by Γ_2_ = 0.07 cm^2^/mJ, the red curve is described by Γ_2_ = 0.7 cm^2^/mJ, and the blue curve is described by Γ_2_ = 7.0 cm^2^/mJ. (**f**) Determination of Γ_4_ by the tail height of the curve for 265 nm and 10 mJ/cm^2^ results, where the green curve is described by Γ_4_ = 2.8 cm^3^/s, the red curve is described by Γ_4_ = 28 cm^3^/s (red circles are experimental results), and the blue curve is described by Γ_4_ = 280 cm^3^/s.

The inactivation ratio [Log(N/N_0_)] at the same D but different Ps and τs are plotted as a function of τ (from 0 to 1000 s) by red circles (10 mJ/cm^2^) and blue circles (5 mJ/cm^2^) as shown in Fig. 2a,b,c (a: 265 nm, b: 280 nm, c: 308 nm). Here, the chosen values of P and τ are listed in Table 1 [(a) 265 nm; 5 mJ/cm^2^, (b) 265 nm; 10 mJ/cm^2^, (c) 280 nm; 5 mJ/cm^2^, (d) 280 nm; 10 mJ/cm^2^, (e) 308 nm; 5 mJ/cm^2^, and (f) 308 nm; 10 mJ/cm^2^].

For 265 nm irradiation and D = 10 mJ/cm^2^ [red circles in Fig. 2a], the reduction of the P by two to three orders of magnitude (Here we compare the ratios obtained for τ≒0 s and τ≒1000 s.) causes the significant reduction of the ratio by approximately one order of magnitude, which suggests that the efficacy of inactivation was approximately 10 times greater (*p*-value < 0.05) for longer τs and a lower P. By lowering the D from 10 mJ/cm^2^ to 5 mJ/cm^2^, as shown in the blue circles of Fig. 2a, similar results were obtained. However, the difference in the ratios at different Ps was less pronounced and the efficacy of inactivation was reduced to approximately 7 times (*p*-value < 0.05).

At the same D, similar results were obtained with 280 nm irradiation as shown in Fig. 2(b) (red circles: 10 mJ/cm^2^ and blue circles: 5 mJ/cm^2^). The difference in the ratios at different Ps was less pronounced. For example, the efficacy of D = 10 mJ/cm^2^ was approximately 7 times greater (*p*-value < 0.05) and that of D = 5 mJ/cm^2^ was approximately 5 times greater (*p*-value < 0.05) for longer τ (τ≒1000 s) and lower P compared to shorter τ (τ ≒0 s) and higher P.

However, at the irradiation wavelength of 308 nm, we could not observe significant reduction of the ratios by changing the P under the same D conditions, as shown in Fig. 2c. For example, the efficacy of D = 10 mJ/cm^2^ (red circles) was approximately 1.3 times greater (*p*-value = 0.19) and that of D = 5 mJ/cm^2^ (blue circles) was approximately 1.2 times greater (*p*-value = 0.23) for longer τ (τ ≒1000 s) and lower P compared to shorter τ (τ≒0 s) and higher P. However, the *p*-values show that there is no statistical difference in the ratio between longer and shorter τs.

The initial CFU was varied between 10^2^ and 10^4^ to examine whether the reduction ratios depended on the number of initial CFU. However, as was similarly observed by Hamamoto et al.^23^, we could not observe significant change of the reduction ratios.

### Stochastic model with two inactivation processes

Target theories with single-hit or multihit models are generally used for the analysis of UV inactivation^24–26^, and the Bunsen-Roscoe law^6^ is the basic principle for the analysis of UV inactivation. However, the large difference in the efficacy of inactivation at the same D but different Ps at a fixed wavelength cannot be explained by these theories. On the other hand, it is generally known that UV radiation generates reactive oxygen species (ROS) and that ROS damage DNA, membranes, and cells^27,28^. Recent results suggest that ROS play an important role in UV inactivation and that for a given D, UV inactivation is more effective for a lower P and longer τs^27,28^.

**Figure 3:**
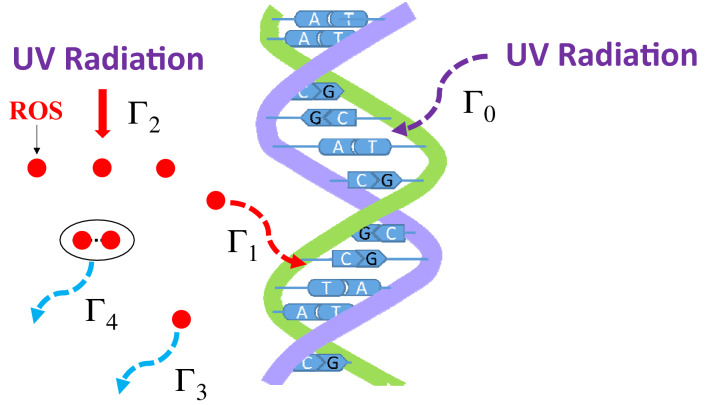
Quantitative model describing DNA damage processes. UV radiation directly causes DNA damage by the formation of thymine dimers or the generation of ROS radicals (red circles) at bacteria by UV radiation, which damages DNA. The rates of DNA damage are described as follows: Γ_0_ (cm^2^/mJ): UV radiation directly causes DNA damage by the formation of thymine dimers, Γ_1_ (cm^3^/s): ROS radicals damage DNA, Γ_2_ (cm^2^/mJ): ROS radicals are generated at the bacteria by UV radiation, Γ_3_ (s^−1^): lifetime of ROS radicals, and Γ_4_ (cm^3^/s): mutual destruction of ROS radicals.

Here, we assume that both ROS and UV radiation cause DNA damage. Figure 3 shows the quantitative model that describes the processes and their rates of DNA damage: (i) Γ_0_ (cm^2^/mJ): the rate at which UV radiation directly causes DNA damage by the formation of thymine dimers^29–33^; (ii) Γ_1_ (cm^3^/s): the rate at which ROS radicals cause damage to DNA; (iii) Γ_2_ (cm^2^/mJ): the rate of generation of ROS radicals at the bacteria by UV radiation; (iv) Γ_3_ (1/s): the lifetime of ROS radicals ^34–37^; and (v) Γ_4_ (cm^3^/s): the rate of mutual destruction of ROS radicals. In this case, the reduction rate of bacterial number N(t) (1/cm^3^) and the generation rate of ROS radicals R(t) (1/cm^3^) as a function of time (0≤t≤τ) can be expressed by the following stochastic differential equations:

1 $$\frac{dN(t)}{dt}=-{\Gamma }_{0}PN\left(t\right)-{\Gamma }_{1}N\left(t\right)R\left(t\right),$$

2 $$\frac{dR(t)}{dt}={\Gamma }_{2}PN\left(t\right)-{\Gamma }_{1}N\left(t\right)R\left(t\right)-{\Gamma }_{3}R\left(t\right)-{\Gamma }_{4}R\left(t\right)R\left(t\right),$$

where P is the irradiance (mW/cm^2^). By solving these two differential equations, both N(t) and R(t) can be described by the variable t with the P as a parameter. By considering the constant D condition such as τ × P = D (mJ/cm^2^) = constant for the solution of the above Eqs. (1) and (2), we can describe the efficacy of inactivation at the same D but different Ps.

Here, we quantitatively describe the procedure for the determination of each rate based on the result of the 10 mJ/cm^2^-D and 265 nm irradiation shown in Fig. 2a as a representative example. We integrate Eq. (1) as follows:

3 $$N\left(\tau \right)={N}_{0}\mathrm{exp}[{-\Gamma }_{0}P\tau -{\Gamma }_{1}{\int }_{0}^{\tau }R\left(t\right)dt].$$

By considering Eq. (3), in the limit of τ→0 with τ × P = D (mJ/cm^2^) where D is constant, Γ_0_ (cm^2^/mJ) can be determined by the value of the Log (N/N_0_)-intercept. We obtained Γ_0_ = 0.22 (cm^2^/mJ). Here, we note that this theoretical curve does not start from (0, 0) but start from (0, −  Γ_0_D/2.3) because P is described as P = D/τ [see Eq. (3)]. Thus, decreasing the D leads to a larger value of the intercept. This tendency agrees with the experimentally observed values of the intercept, as shown in Fig. 2a.

Other parameters, such as Γ_1_, Γ_2_, and Γ_4_, can be determined by the curve characteristics, as shown in Fig. 2d–f. For example, the value of Γ_1_ is reflected in the initial slope of the curve, as shown in Fig. 2d, where the green curve is described by Γ_1_ = 2 × 10^−4^ (cm^3^/s), the red curve is described by Γ_1_ = 2 × 10^−3^ (cm^3^/s) (red circles are experimental results), and the blue curve is described by Γ_1_ = 2 × 10^−2^ (cm^3^/s); hence we choose Γ_1_ = 2 × 10^−3^ (cm^3^/s). Next, the value of Γ_2_ is determined by the tail slope of the curve, as shown in Fig. 2e, where the green curve is described by Γ_2_ = 0.07 (cm^2^/mJ), the red curve is described by Γ_2_ = 0.7 (cm^2^/mJ) (red circles are experimental results), and the blue curve is described by Γ_2_ = 7.0 (cm^2^/mJ); and we choose Γ_2_ = 0.7 (cm^2^/mJ). The parameter Γ_4_ is determined by adjusting the tail height of the curve, as shown in Fig. 2f, where the green curve is described by Γ_4_ = 2.8 (cm^3^/s), the red curve is described by Γ_4_ = 28 (cm^3^/s) (red circles are experimental results), and the blue curve is described by Γ_4_ = 280 (cm^3^/s); and we choose Γ_4_ = 28 (cm^3^/s).

The lifetime of ROS is correlated with Γ_3_ and Γ_4_, which does not depend on the irradiation wavelength. After the determination of Γ_4_, the parameter Γ_3_ is determined to be Γ_3_ = 1 (1/s). The lifetime determined for Γ_3_ is likely to be a reasonable value because it agrees well with previously reported values^34,35^. Notably, by using these parameters of Γ_0_ to Γ_4_, which are determined based on the results of 265 nm and 10 mJ/cm^2^, we can draw theoretical curves of 265 nm for various dose conditions. The blue curve shown in Fig. 2a was drawn for 265 nm and D = 5 mJ/cm^2^ condition using the same Γ_0_ to Γ_4_.

Figure 2a,b,c show the experimental plots (solid circles) and theoretically fitted curves (solid curves) obtained by the above fitting procedure for irradiation wavelengths of 265 nm [Fig. 2a], 280 nm [Fig. 2b], and 308 nm [Fig. 2c], where the red-circles and -curves represent a D of 10 mJ/cm^2^, and the blue-circles and -curves represent a D of 5 mJ/cm^2^, respectively. The values of τs to fit the curves for each irradiation wavelength are denoted in Table 2. The theoretical curves explain the inactivation behaviour well as a function of the τ under different irradiation wavelengths and D conditions. Although the assumption that ROS is involved in the DNA damage^27,28^ is an issue to be addressed in the future, we consider that the stochastic model presented here explains well not only the present results but also the wide range of inactivation rate constants previously reported^12–19^.

**Table 2:** The values used to obtain the theoretically fitted curves shown in Fig. 2a,b,c, derived from Eqs. (1) and (2). Here, Γ_0_ (cm^2^/mJ) is the rate of UV radiation directly causing DNA damage by the formation of thymine dimers; Γ_1_ (cm^3^/s) is the rate of ROS radicals causing damage to DNA; Γ_2_ (cm^2^/mJ) is the rate of generation of ROS radicals at bacteria by UV radiation; Γ_3_ (1/s) is the lifetime of ROS radicals; and Γ_4_ (cm^3^/s) is the mutual destruction rate of ROS radicals.

Λ (nm)	Γ_0_	Γ_1_	Γ_2_	Γ_3_	Γ_4_
265	0.22	2 × 10^–3^	0.7	1	28
280	0.22	2 × 10^–3^	0.5	1	28
308	2 × 10^–3^	6.5 × 10^–5^	0.5	1	28

## Discussion

**Figure 4:**
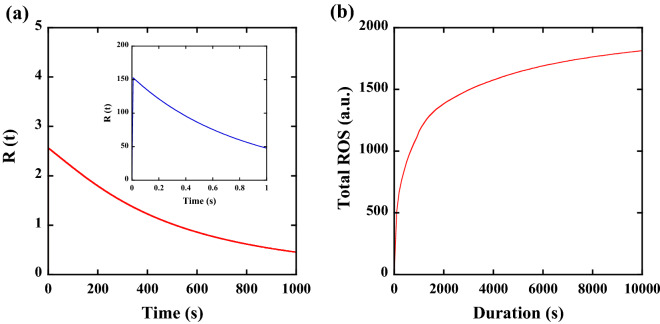
(**a**) Temporal behaviour of R(t) obtained at 0.01 mW/cm^2^ with 1000 s (red curve) or 10 mW/cm^2^ with 1 s (blue curve in the inset) at the irradiation wavelength of 265 nm. (**b**) Total amount of ROS as a function of irradiation duration at the same dose (265 nm, 10 mJ/cm^2^).

It is interesting to show the difference in the amount of ROS generated by UV irradiation when the D is constant but the P is different. Figure 4a shows the theoretical temporal behaviour of R(t) obtained at 0.01 mW/cm^2^ with 1000 s (red curve) or 10 mW/cm^2^ with 1 s (blue curve shown in the inset) at the irradiation wavelength of 265 nm. The temporal behaviour of both R(t) shows similar curve characteristics. A notable point is the difference in the peak value; although the P is varied by a factor of 1000, the obtained peak value varies by a factor of only 60, such as 150 at 10 mW/cm^2^ and 2.5 at 0.01 mW/cm^2^. This difference originates from the Γ_4_R(t)R(t) nonlinear term, which implies that the high-density ROS state is unstable and mutual destruction of ROS occurs^38^. The difference in the peak value leads to the difference in the total amount of ROS at the same D. Figure 4b shows the total amount of ROS as a function of τ at the same D (265 nm, 10 mJ/cm^2^). Due to the long lifetime of ROS (Γ_3_) and the nonlinear term (Γ_4_), weaker irradiance with longer duration generates a larger amount of ROS. We consider this difference in the amount of ROS as a function of τ to be the physical and chemical mechanism that explains the large difference in the efficacy of inactivation at the same D.

We could not observe a significant change in efficacy versus P at the irradiation wavelength of 308 nm. This result is similar to the results observed by Oguma et al.^16^ but is contrary to the findings of Pousty et al.^27^. The reason for this is not clearly understood; however, we consider this difference in efficacy to originate from the difference in the strain: where we used a simple O1 strain, Oguma et al. used the K12 IFO 3301 strain^16^, and Pousty et al. used the MG1665 strain^27^. To clarify this issue, other strains of *E. coli* bacteria are now under investigation. The fact that we could not observe a significant reduction in efficacy at the irradiation wavelength of 308 nm is likely to suggest that the mechanism of ROS generation by UV light correlates with the absorption spectrum of DNA^19,39,40^ and/or protein^41–46^ species.

The tendency in the behaviour of the P and the efficacy obtained in this work seems to be consistent with the previous studies^9,13,47–49^. For a smaller P, a higher inactivation rate constant was reported. For example, at a 265 nm irradiation wavelength, for the *E. coli* K12 29425 strain, the reported reduction rate is Log (N/N_0_) = − 1.5 for 5 mJ/cm^2^ and − 2.5 for 10 mJ/cm^2^ in the smaller P region (0.030–0.060 mW/cm^2^)^47^; however, in the larger P region (0.19–0.55 mW/cm^2^), the reduction rate decreases as Log (N/N_0_) = − 1 for 5 mJ/cm^2^ and − 2 for 10 mJ/cm^2^^48^. A very similar tendency was reported for *E. coli* CGMCC 1.3373, and the reported reduction rate is Log (N/N_0_) =  − 1.5 for 5 mJ/cm^2^ and − 4.5 for 10 mJ/cm^2^ in the smaller P region (0.05 mW/cm^2^)^13^; however, in the larger P region (0.384 mW/cm^2^), the reduction rate decreases as Log (N/N_0_) =  − 1 for 5 mJ/cm^2^ and − 3 for 10 mJ/cm^2^^49^.

We note here that the processes of birth and death of *E. coli* were not considered in this analysis, because the inactivation assays were performed under a stationary phase [*E. coli* in a normal saline solution (0.9% NaCl)]. However, when we perform the UV inactivation assays under a well-nourished phase (logarithmic phase), we have to consider the duplication time, because *E. coli* in the well-nourished state divides every 20 min^50^. In this case, it is necessary to introduce the birth and death processes that show this proliferation effect into this stochastic model.

## Conclusions

In this paper, we have clarified the significant difference in the efficacy of inactivation of *E. coli* under the same D but different Ps and τs at a fixed wavelength. Although thymine dimer production and ROS production were not confirmed experimentally, we believe that during the UV inactivation process, in addition to the formation of thymine dimers in DNA, another factor, such as ROS, played an important role in the inactivation of bacteria. The efficacy of inactivation by ROS depends on the P, while the formation of thymine dimers depends on the D. To prove that ROS play a role in inactivation, it is necessary to quantify and measure the amount of ROS by UV irradiation.

Various values of the inactivation rate constant for the UV inactivation of a bacterium and/or virus have been reported, even when the same light source and irradiation wavelength are used^9–11^. One reason for this discrepancy might be the difference in strains and their environments of the bacteria. However, according to the experimental and theoretical results obtained here, it is likely that the UV inactivation rate constants reported until now are composed of mixed values of two inactivation processes that depend on the magnitude of P. Therefore, the inactivation rate constants reported in the literature vary widely, even when the same wavelength of irradiation as well as the same types and strains of bacteria and viruses were used. To validate the applicability of the model obtained here to a broader set of pathogens, inactivation experiments combining short-term irradiation with high P are necessary to determine the actual UV inactivation rate constants.

The amount of UV radiation to which the human body can be subjected is limited by a threshold limit value (TLV) for each wavelength based on the American Conference of Governmental Industrial Hygienists (ACGIH)-TLV booklet^51^. The results of the present study show that for the same D, inactivation at a lower P and longer τ is more efficient than inactivation at a higher P and shorter τ. The effectiveness of prolonged UV irradiation at a lower P can reduce the D and the risk to the human body. Thus, we consider this information to be useful for the future sterilization of large spaces such as hospital rooms using UV light. To achieve such lighting technologies with germicidal effects, it is necessary to investigate the effect of P under the same D conditions on various types of pathogenic bacteria and viruses.

## Data Availability

The datasets used and/or analysed in the current study are available from the corresponding author on reasonable request.
